# Structural and biochemical insights into HCV envelope proteins for germline-targeting vaccines

**DOI:** 10.1371/journal.ppat.1014651

**Published:** 2026-10-12

**Authors:** ItaiG. Yechezkel, Hila E. Maimon, Ariel Tennenhouse, Fang Chen, Haneen Tarabih, Jenna Weisz, Rina Fraenkel, Sarel J. Fleishman, Mansun Law, Netanel Tzarum

**Affiliations:** 1 Department of Biological Chemistry, Alexander Silberman Institute of Life Sciences, Faculty of Mathematics & Science, The Hebrew University of Jerusalem, Jerusalem, Israel; 2 Department of Biomolecular Sciences, Weizmann Institute of Science, Rehovot, Israel; 3 Department of Immunology and Microbiology, The Scripps Research Institute, La Jolla, California, United States of America; 4 The Center for Nanoscience and Nanotechnology, The Hebrew University of Jerusalem, Jerusalem, Israel; The University of Chicago, UNITED STATES OF AMERICA

## Abstract

Despite effective antivirals, Hepatitis C remains a global health burden, reflecting the difficulty of eliciting broadly neutralizing antibodies (bnAbs). For the Hepatitis C virus, bnAb responses are biased toward V_H_1-69-encoded Abs targeting a conserved epitope on the E2 glycoprotein, suggesting that effective immunogens must engage the corresponding germline (GL) B cell receptors. However, the structural basis of GL recognition remains unclear. Here, we show that GL-reverted V_H_1-69 bnAbs recognize the E2 core domain in a manner closely resembling mature Abs binding. High-resolution structures reveal a conserved mode of engagement, indicating that key epitope features are accessible prior to affinity maturation. Guided by these insights, we engineered E2 variants to enhance GL Ab interactions and stability. Although these designs increased affinity, they did not improve engagement of naïve B cells, highlighting a key constraint in GL-targeting strategies. Together, our findings define principles governing GL recognition and inform the rational design of an HCV vaccine.

## Author summary

Hepatitis C virus (HCV) infects millions of people worldwide and remains a leading cause of chronic liver disease. Although highly effective antiviral drugs can cure infection, they do not prevent new infections, making the development of a preventive vaccine a global priority. One promising strategy is to stimulate rare naïve B cells that can develop into broadly neutralizing antibody-producing cells capable of recognizing many different HCV strains. In this study, we determined how putative antibody precursors recognize a conserved region of the HCV envelope protein at atomic resolution. Using this structural information, we engineered modified viral proteins designed to improve recognition by these precursor antibodies. While the engineered proteins showed binding in biochemical assays, they did not increase recognition of naïve B cells. These findings reveal that improving antibody affinity alone is insufficient to enhance the earliest stages of the immune response. Our work provides new insights into how broadly neutralizing antibodies develop and identifies important considerations for designing next-generation vaccines against HCV and other rapidly evolving viruses.

## Introduction

Hepatitis C virus (HCV) infects approximately 50 million people worldwide [1] and remains a leading cause of chronic liver disease and hepatocellular carcinoma [1–3]. Although direct-acting antivirals cure more than 95% of infections, they do not prevent reinfection [4,5], and challenges including asymptomatic infection and delayed diagnosis continue to hinder global elimination efforts [6]. These limitations underscore the need for an effective prophylactic vaccine.

HCV is an enveloped, positive-sense, single-stranded RNA virus that belongs to the *Hepacivirus* genus within the *Hepaciviridae* family [7–9]. A major challenge in developing an HCV vaccine is the virus’s extreme genetic diversity [10,11], which is currently classified into eight main genotypes and over 90 subtypes [7,12]. The envelope (Env) glycoproteins E1 and E2 form a noncovalent heterodimer on the virion surface and facilitate cell entry. The E2 envelope glycoprotein mediates receptor binding and is the principal target of broadly neutralizing antibodies (bnAbs). However, extensive sequence variability and glycan shielding enable immune escape while limiting access to conserved epitopes (Fig 1A, 1B) [13,14].

**Fig 1:**
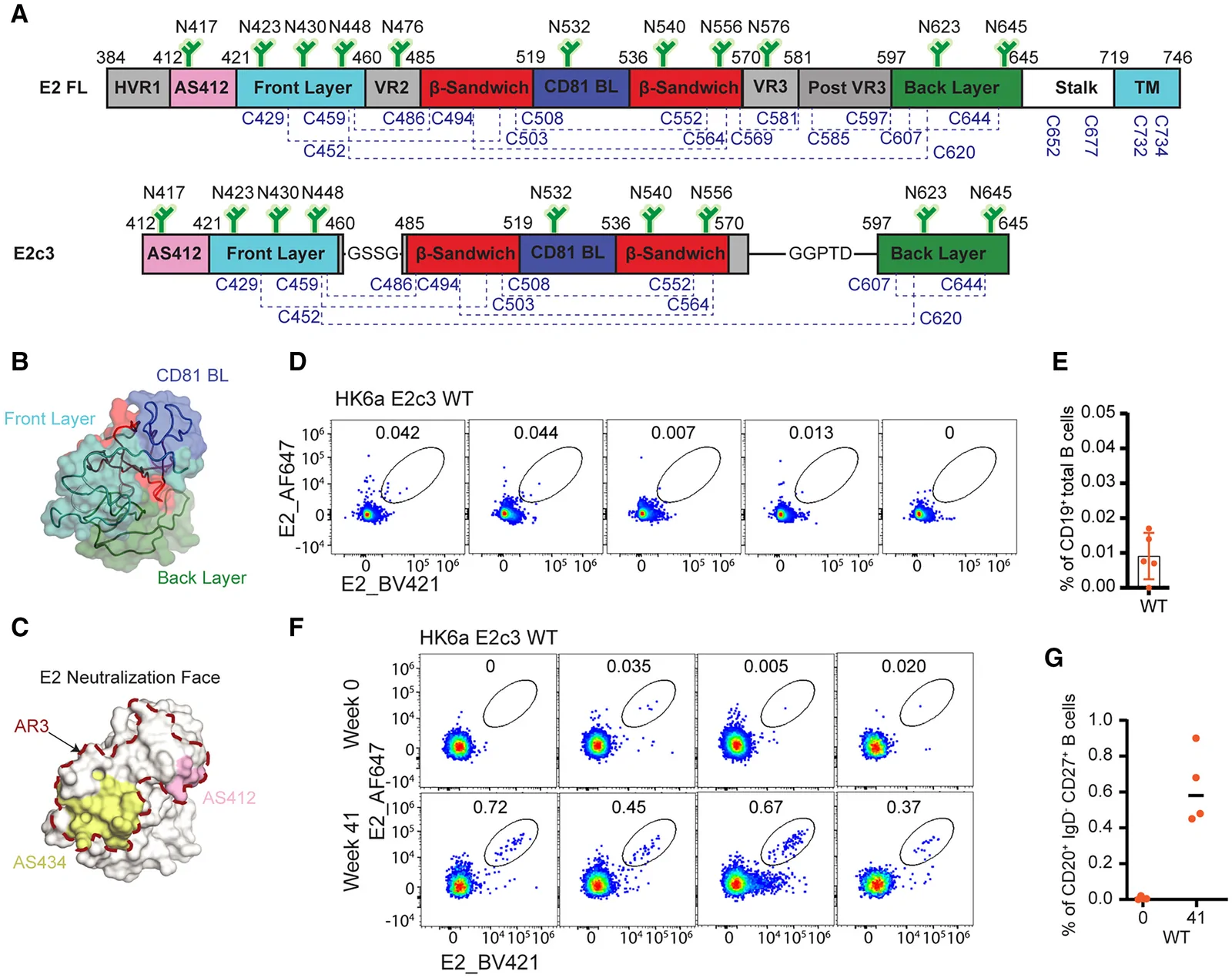
Detection of E2-specific B cells in normal blood donors and NHPs immunized with HCV-1 E1E2. **(A)** Schematic representation of the E2 protein with disulfide bridges shown as blue dashed lines and N-Linked glycosylations as green trees. **(B)** The structure of H77 E2c (PDB: 4MWF) with the molecular surface color-coded as in Fig 1A. **(C)** E2 neutralization face, mapped onto the H77 E2c surface. AR3 is marked in a dashed line, and AS412 and AS434 are colored in pink and yellow, respectively. **(D)** Flow cytometry staining of E2-specific B cells. Cells were gated on CD3^-^ CD19^+^ CD10^-^. **(E)** Frequency of E2-specific B cells in 5 Normal blood donors. **(F)** Flow cytometry staining of E2-specific B cells from NHPs. Cells were gated on CD20 + IgD-CD27 + . Week 0, pre-immunization; week 41, 1 week after the 4^th^ immunization. **(G)** Frequency of E2-specific B cells in blood samples of 4 NHPs.

Previous failures in clinical vaccination trials [15–18] have underscored the need to engineer envelope-derived antigens that can induce high-titer bnAb responses. bnAbs isolated from human and animal models [19,20] have provided critical insights into highly conserved epitopes on the HCV E1 and E2 glycoproteins. Epitope mapping studies reveal that most cross-neutralizing sites, such as antigenic region 3 (AR3), overlap with the CD81 receptor binding site and are highly conserved (Fig 1C) [21,22], likely reflecting strong evolutionary constraints on this region, underscoring their relevance for vaccine design. Over the past fifteen years, X-ray crystallography studies of E2-Ab complexes and cryo-electron microscopy of E1E2-Ab complexes have further defined the antigenic landscape of E2 [9,23,24]. These analyses revealed a conserved, conformationally discontinuous, and predominantly hydrophobic surface, termed the E2 neutralization face, that remains accessible to bnAbs despite extensive N-linked glycosylation [25]. This region comprises the front layer and the CD81-binding loop (CD81 BL) (Fig 1B, 1C) and encompasses the principal neutralization site AR3.

AR3 consists of a cluster of discontinuous epitopes and is targeted by numerous highly potent cross-genotype bnAbs. Genetic analysis indicates that AR3-specific bnAbs are predominantly, though not exclusively [26,27], encoded by the IGHV1–69 (V_H_1-69) heavy-chain variable germline (GL) gene family [28–34]. Shared features across all these V_H_1-69 derived Abs include an atypically hydrophobic heavy chain (HC) complementarity-determining region 2 (CDRH2) [35], an elongated CDRH3 loop of 15–22 a.a. (compared with 9–15 a.a. for healthy donors) [36,37], HC-focused interactions [22], and relatively low levels of somatic hypermutation (SHM) [22,37–40]. Although they share these features, the E2-bnAb complexes demonstrate different modes of binding, which allows V_H_1-69 encoded bnAbs to be clustered into sub-groups based on their angle of approach to E2 and differences in the footprint on E2 [9,41]. Furthermore, research on B-cell responses in rhesus macaques vaccinated with the Chiron E1E2 vaccine indicated that AR3 can be targeted by bnAbs encoded by V_H_1-69-equivalent GL genes [41], with structural analyses showing binding features similar to those of human AR3-directed bnAbs [27,42,43].

Together, structural and genetic analyses of V_H_1-69-derived bnAbs show that, despite substantial plasticity within AR3, this region can support interactions with bnAbs that vary in CDR sequence and length. This versatility makes AR3 an attractive target for the design of GL-targeted vaccines. The concept of germline-targeting immunogens was pioneered in HIV-1 vaccine development to activate VRC01-class precursor B cells [44–46] and has since been extended to influenza virus [47], SARS-CoV-2 [48], and respiratory syncytial virus [49], demonstrating its broader applicability [46,47].

The strong bias toward V_H_1-69-derived AR3-targeted Abs provides an enticing rationale for GL-targeted vaccine design in HCV, particularly because previous studies demonstrated that V_H_1-69 GL-reverted Abs retain measurable binding to the E2 glycoprotein while exhibiting markedly reduced neutralization potency and breadth compared with their mature counterparts [37,40,43,50]. These findings suggest that productive engagement of V_H_1-69 GL B-cell receptors (BCRs) is an essential first step toward eliciting bnAbs, but that affinity maturation is required to achieve potent, broad virus neutralization. Here, we combined structural biology, biochemistry, and protein engineering to elucidate how germline-reverted AR3-targeting Abs recognize the HCV E2 glycoprotein. Guided by these structures, we designed E2 variants to enhance GL Ab engagement. Although these variants maintained binding they failed to increase naïve B-cell recognition, revealing an important limitation of affinity-based GL-targeting strategies. Together, our findings provide mechanistic insights into V_H_1-69-mediated recognition of HCV E2 and establish design principles for next-generation HCV vaccine immunogens.

## Results

### Limited engagement of naïve B cells to HCV E2

To assess the baseline immunogenicity of wt E2, we quantified the frequency of E2-specific B cells within the naïve B cell repertoire of five HCV-negative normal blood donors. The binding of primary B cells to the HK6a E2c3 antigen (E2 residues 412–645 with an internal truncation of VR2, VR3, and the removal of N448 and N576 glycosylation sites; Fig 1A and methods) was evaluated by fluorescence-activated cell sorting (FACS). These analyses revealed minimal binding, with antigen-specific populations consistently representing fewer than 0.02% of total circulating B cells (Fig 1D,1E). We next examined the frequency of E2-reactive B cells in rhesus macaque samples from animals vaccinated with HCV-1 E1E2 mRNA or protein antigens, which were previously shown to elicit V_H_1-69-equivalent bnAb responses, although at low circulating levels [41]. Consistent with the normal blood donors’ data, naïve B cells from pre-immune rhesus macaques (week 0) exhibited limited binding to HK6a E2c3 (average 0.02%; Fig 1F,1G). In contrast, 1 week after the 4^th^ immunization (week 41), a substantial increase in HK6a E2c3-specific memory B cells (CD20 ⁺ IgD^−^ CD27⁺) was observed, with frequencies rising to approximately 0.6% (Fig 1F,1G). These data suggest that wt E2c3 recruits GL-encoded BCRs poorly.

### Reversion to GL Abs compromises the efficacy of AR3-targeted V_H_1-69 Abs

An in-depth functional and structural analysis of how GL Ab precursors recognize their target antigens can provide essential insights into bnAb induction and enable the design of improved antigens [41]. To this end, we chose four AR3-targeting, V_H_1-69-encoded Abs that we previously used to structurally define the E2 neutralization face: the bnAbs AR3A and AR3C, and the nAbs U1 and 212.1.1. These Abs engage AR3 through two distinct binding modes [9] (S1 Fig), providing a framework for studying how various GL precursors recognize E2. We used the IMGT V-QUEST program [51,52] to infer GL sequences of the HC (V_H_, D_H_, and J_H_) (S2A Fig) [due to the HC dominance, the LC was not reverted] [37,38]. The inferred GL precursors and variants with only the V_H_ gene reverted were synthesized for further analysis (Ab_GL_ and Ab_1–69_, respectively). Note that our previous study indicated low binding affinity of AR3A_GL_ and AR3C_1-69_ to H77 and HK6a E2c3 [37], therefore, these Abs were excluded from this study. Sequence alignment showed high overall homology between the inferred GL and mature Abs, with 78.12-88.8% amino acid identity (S2B Fig). Except for 212.1.1, the most notable differences were localized to CDRH1 (33–50% identity), while CDRH2 showed higher conservation (65–76% identity), including conservation of key hydrophobic residues thought to be involved in interactions with the hydrophobic E2 neutralization face (Fig 2A).

**Fig 2:**
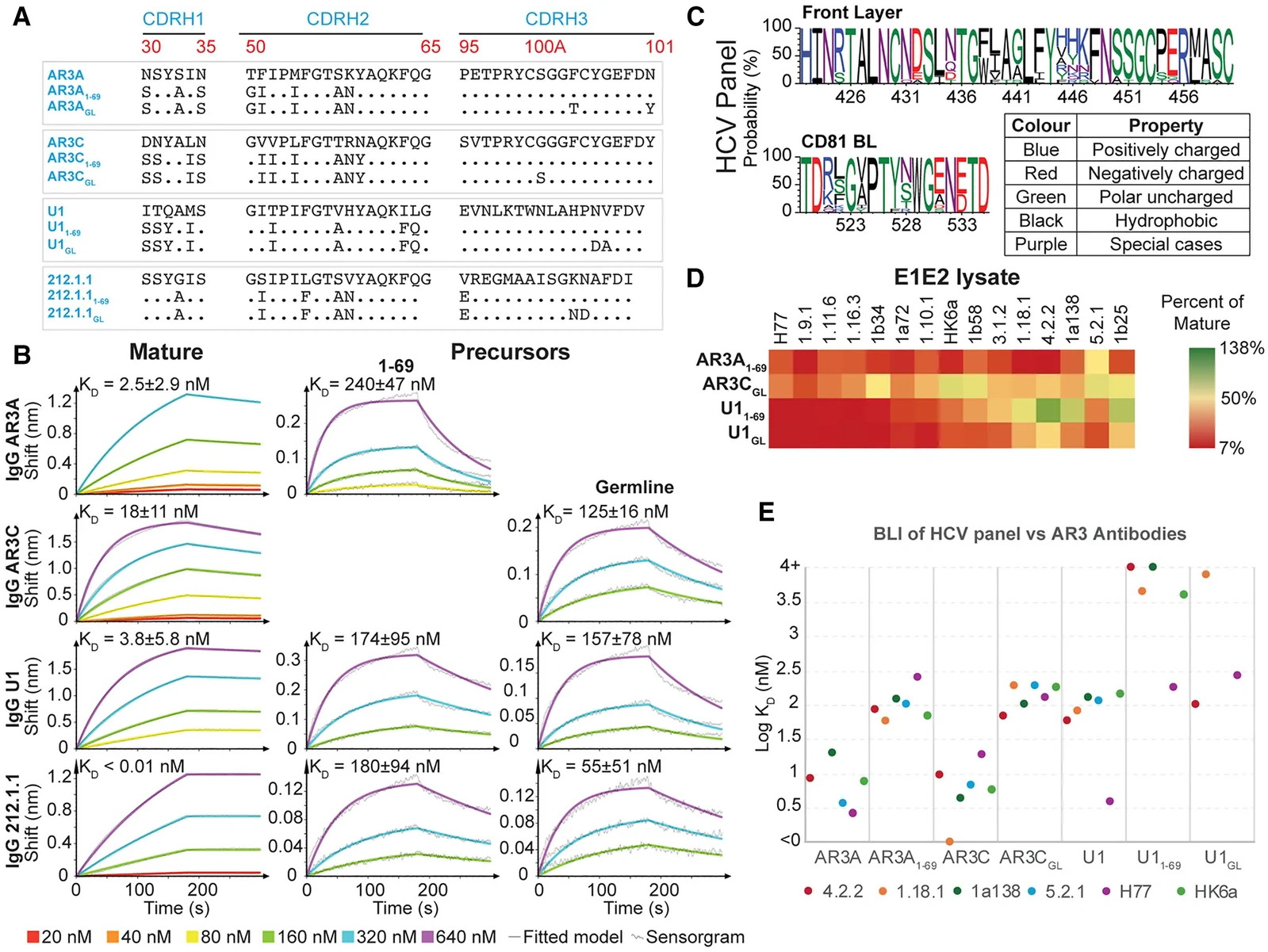
Biochemical characterization of the GL-reverted AR3-targeting Abs. **(A)** Sequence alignment of the HC CDRs of the mature and inferred GL Abs (inferred GL and reversion of only the VH gene to the V_H_1-69 gene sequence) of the AR3-targeting nAbs AR3A, AR3C, U1, and 212.1.1. **(B)** BLI binding kinetics of H77 E2c3 to the mature and GL-reverted Abs. Sensorgrams were obtained from three to six two-fold titration series starting at 640 nM, and K_D_ values were calculated using a 1:1 global fitting model (solid line). **(C)** Sequence LOGO graph presenting the conservation of the front layers and the CD81 BL region of the HCV reference panel. **(D)** ELISA-based cross-reactivity analysis of mature and GL-reverted Abs against E1E2 cell lysate from a reference panel of HCV isolates. Data are presented as a heat map, where binding of GL-reverted Abs is expressed as a percentage of the corresponding mature Ab for each isolate. The color scale is shown on the right. **(E)** Binding affinity (K_D_) summary of mature and GL-reverted Abs against purified E2c3 of UKNP 4.2.1, UKNP 1.18.1, UKNP 5.2.1, 1a138, HK6a, and H77 isolates determined by BLI.

To measure antigen recognition by GL-reverted Abs, we expressed both mature and GL-reverted Abs as IgGs and used biolayer interferometry (BLI) to determine binding kinetics to H77 E2c3 (Fig 2B). Mature Abs bound E2 with high affinity, with dissociation constants (K_D_) ranging from <0.01 nM for 212.1.1 to 18 nM for AR3C. Conversely, GL-reverted variants showed significantly lower affinities, with K_D_ values from 55 nM (212.1.1_GL_) to 240 nM (AR3A_1-69_). The degree of affinity reduction varied widely among the Abs, from a modest 5–10-fold decrease for AR3A, AR3C, and U1 to more than 1,000-fold for 212.1.1. This reduced binding was mainly due to faster dissociation rates (k_off_), emphasizing the importance of SHM in stabilizing the E2-Ab interaction and indicating that GLs have limited ability to maintain stable engagement with E2.

We have previously demonstrated, using a cross-genotype HCV cell culture (HCVcc) panel, that GL reversion of AR3A and AR3C markedly reduced both neutralization potency and breadth, despite retaining measurable binding to E2 [37]. Although limited neutralization was observed against a small subset of isolates, primarily genotypes 5a and 6a, most GL-reverted Abs exhibited little or no neutralizing activity, precluding reliable IC_50_ determination. These findings suggested that affinity maturation is required for the acquisition of broad and potent neutralizing activity. To verify these observations, we evaluated the neutralizing activity of mature and GL-reverted AR3A, AR3C, and U1 Abs using H77 HCV pseudoparticles (HCVpp). Consistent with our previous HCVcc results, GL reversion led to a marked reduction in neutralization potency (S2G Fig).

To evaluate the impact of the SHMs on binding breadth and affinity, we compared the binding profiles of mature and reverted GL Abs against native E1E2s from an antigenically diverse HCV reference panel [53] using enzyme-linked immunosorbent assays (ELISAs). Sequence alignment of the front layer and the CD81 BL regions of the reference panel shows overall high conservation (Fig 2C). Binding was assessed for mature IgGs (AR3A, AR3C, U1) alongside their respective precursors (AR3A_1-69_, AR3C_GL_, U1_1-69_, and U1_GL_) (Fig 2D). Initial screening against the 15-panel isolates revealed that, while for some isolates, the precursor Abs showed binding levels more comparable to their mature Ab counterparts, the overall binding magnitude of both mature and GL Abs was lower than that of H77 and HK6a. Consistent with the negligible binding observed in our primary B cell FACS assays, HK6a E1E2 (Genotype 6a) showed a significant loss of recognition upon GL reversion, strengthening the importance of the SHMs in maintaining affinity.

We identified a subset of isolates, including 1b25, UKNP 5.2.1, 1a138, UKNP 4.2.2, and UKNP 1.18.1, that showed the most consistent binding between mature and GL Abs. This suggests that, although the AR3 Abs generally rely on somatic maturation for high-affinity binding, some HCV isolates naturally tolerate GL-encoded Abs more readily, thereby increasing their potential for future vaccine development. To investigate this further, we expressed and purified E2c3 constructs for these isolates (noting that 1b25 E2c3 failed to express) (S2C Fig). Subsequent ELISA analysis with these purified antigens confirmed that the isolates maintained comparable binding to the mature and GL Abs, though, as before, the absolute binding was considerably lower than that observed for H77 and HK6a (S2D Fig).

To quantify these interactions, we measured the binding kinetics of the purified E2c3 subset, HK6a, and H77 against mature and GL Abs using BLI (2E and S2E, S2F Fig). Both AR3A and AR3C exhibited high affinities, with K_D_ values ranging from <0.01 nM (AR3C against UKNP 1.18.1) to 20.1 nM (AR3A against 1a138). The reverted counterpart, AR3A_1-69_, showed consistently higher affinity for the subset isolates (K_D_ values ranging from 59.3 - 127 nM) compared to H77 (240 nM), and HK6a (65.2 nM), suggesting these isolates could serve as viable initial scaffolds for vaccine design. Notably, UKNP 5.2.1 exhibited an approximately 2-fold increase in affinity for both reverted AR3A Abs compared with H77. However, in contrast to the previous ELISA results (Fig 2D and S2D Fig), AR3C_GL_ showed reduced but measurable affinities that were more consistent with the H77 baseline (125 nM) or the HK6a baseline (173 nM), ranging from 71.6 nM (against UKNP 4.2.2) to 193 nM (against UKNP 1.18.1) (Fig 2E). U1 also generally demonstrated lower affinities for the subset isolates, with the reverted U1 Abs showing a particularly sharp decline in binding. Taken together, these results support the continued use of H77 or HK6a as practical template antigens for vaccine design, as their GL Ab binding generally falls within the range observed for genetically diverse circulating HCV isolates.

### High-resolution crystal structures of GL reverted Fab-E2c3 antigen complexes

To provide a high-resolution structural basis for the observed GL reactivity, we determined the crystal structures of the HK6a E2c3 core in complex with the Fab domain of four reverted Abs: AR3A_1-69_, AR3C_GL_, U1_1–69_, and U1_GL_ at 2.0, 1.9, 2.7, and 3.3 Å resolution, respectively (see S1 Table for full details). Across all four structures, the electron density maps clearly defined the Ab-antigen interfaces, allowing for a detailed atomic comparison of GL versus mature antigen engagement.

Overall, no substantial redistribution of the angle of approach (S3A Fig) or change in the CDRs’ position and conformation was observed (Fig 3A), and HC dominance was preserved, consistent with the canonical binding mode of V_H_1-69 encoded Abs (Fig 3B). Buried surface area analysis of the E2c3-Fab interfaces, including contributions from the full Fab, individual HC and LC, and the HC CDR loops (CDRH1–3), revealed only modest differences between GL reverted and mature Abs complexes (Fig 3A, 3B). Notably, however, AR3C_GL_ showed no measurable contribution to buried surface area from CDRH2, indicating an apparent loss of CDRH2-E2c3 interactions that are prominent in the corresponding mature complex (S3B Fig). Given the well-known role of the hydrophobic CDRH2 motif in V_H_1-69 mediated recognition [38], this observation was unexpected. Therefore, we assume that this difference results from crystal packing constraints rather than from a biologically relevant change in binding, a point further addressed in the discussion.

**Fig 3:**
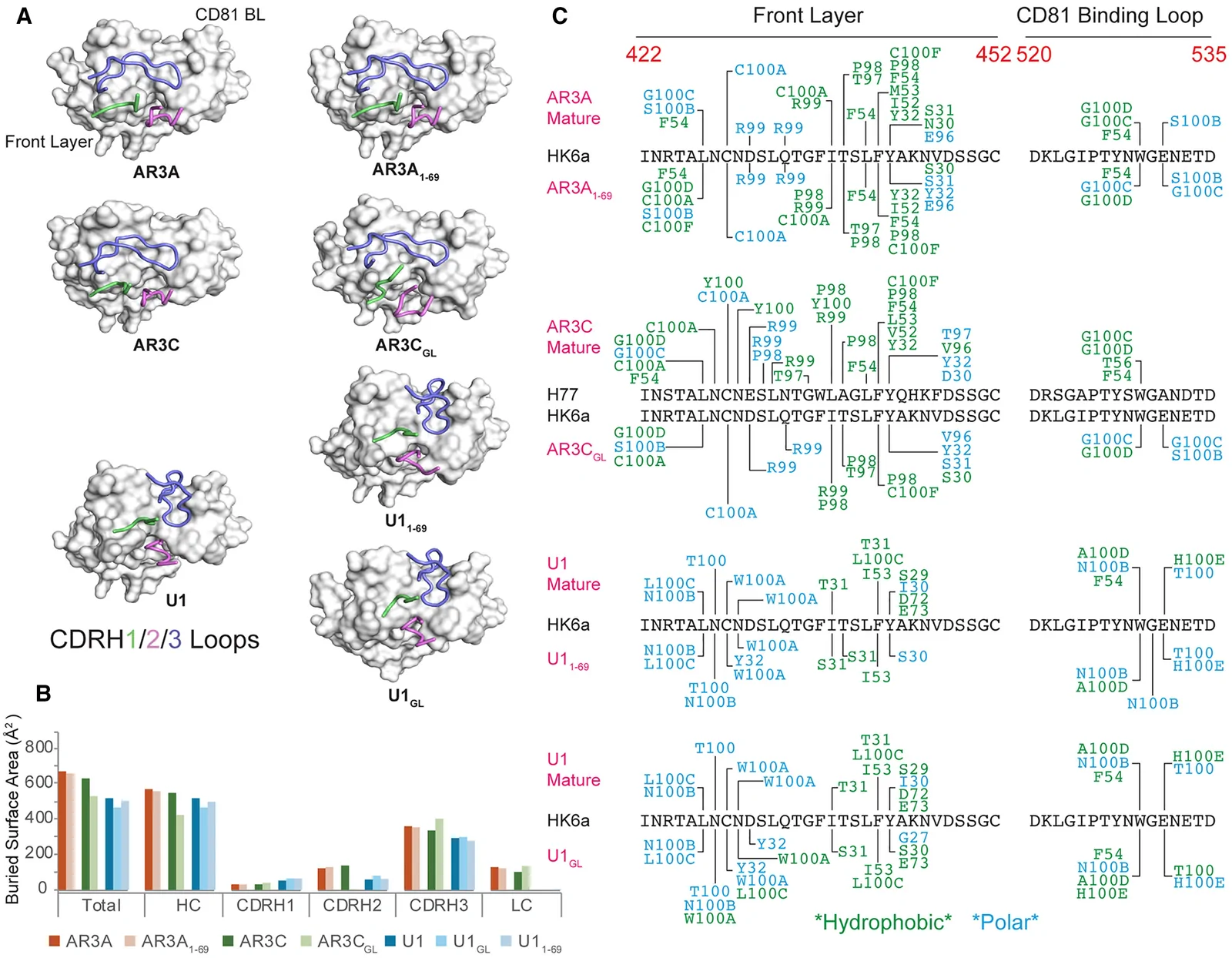
Structural characterization of mature and GL-reverted Abs. **(A)** The position of the CDRH1, CDRH2, and CDRH3 in the E2c3-Fab structures. The E2c3 is shown in surface presentation. The CDRH1, CDRH2, and CDRH3 are shown as cartoons, colored green, violet, and blue, respectively. **(B)** Comparison of buried surface area between mature complex structures (PDB IDs: 4MWF, 6BKB, 6WO3) and their GL-reverted structures. Buried surface area was calculated for the total Ab-antigen interface, the individual HC and LC, and the specific CDR loops of the HC. **(C)** Schematic contact map summarizing interactions between E2c3 and mature and GL-reverted Abs. The E2c3 front layer and CD81 BL primary sequences are displayed with interactions formed by mature Ab indicated above the sequence and those formed by the precursor Abs indicated below. Hydrophobic interactions are shown in green, and polar interactions are shown in blue.

Next, we mapped the interactions between E2c3 and Abs, focusing on the front layer (a.a. 422–452) and the CD81-binding loop (a.a. 520–535), and compared them with their mature counterparts (3C, and S3C Fig). The majority of the interactions were maintained, including contacts mediated by residues that undergo somatic mutation during affinity maturation (e.g., the amino acid at position 30 of the HC, S3C Fig). These observations suggest that GL precursors engage the antigen in a manner that closely resembles that of mature Abs.

### Structure-based design and kinetic validation of optimized E2c3 immunogens

Leveraging structural insights from both GL reverted Ab-E2c3 complexes and mature Ab-E2 complexes, we aimed to engineer an E2 antigen with enhanced affinity for GL-reverted Abs. To this end, we employed two design protocols (see Materials and methods) to improve protein stability. In the first protocol, we utilized the automated design method FuncLib [54] to redesign E2 to optimize the Ab-antigen interface, thereby directly strengthening interactions with GL-reverted Abs. FuncLib integrates evolutionary and Rosetta structure-based calculations to identify stable mutation combinations. The method begins with a phylogenetic analysis to restrict allowable mutations at selected positions to those commonly observed in natural homologs, and then models the allowed point mutations in Rosetta to eliminate highly destabilizing variants. FuncLib subsequently enumerates and evaluates all permitted mutation combinations using atomistic Rosetta design, followed by relaxation and energy-based ranking.

Given the pronounced conformational flexibility of the E2 front layer [9,25], we introduced and successfully expressed five core-stabilization designs (CD1.0-5.0) (S4A Fig). However, upon purification, these variants eluted from the SEC column as broad, poorly defined peaks, indicating conformational heterogeneity and/or partial misfolding rather than the compact, monodisperse profile observed for the wild-type (wt) E2c3. Therefore, we designed two additional iterative designs (CD3.1 and CD3.2) based on CD3, given the shared mutations across all designs. CD3.1 and CD3.2 were expressed and eluted from an SEC column at the same volume as E2c3 wt (S4B Fig). Subsequently, CD3.2 was used for downstream BLI and FACS experiments (Fig 4A, and S4C, S4D Fig). We also designed five interface-optimized variants (ID1.0-5.0) along with six iterative designs (S4A, S4B Fig). We successfully expressed seven of these designs, but only five retained native folding as confirmed by SEC. Following affinity screening, ID3.0 was identified as the top candidate from this group (Fig 4A, and S4A Fig)

**Fig 4:**
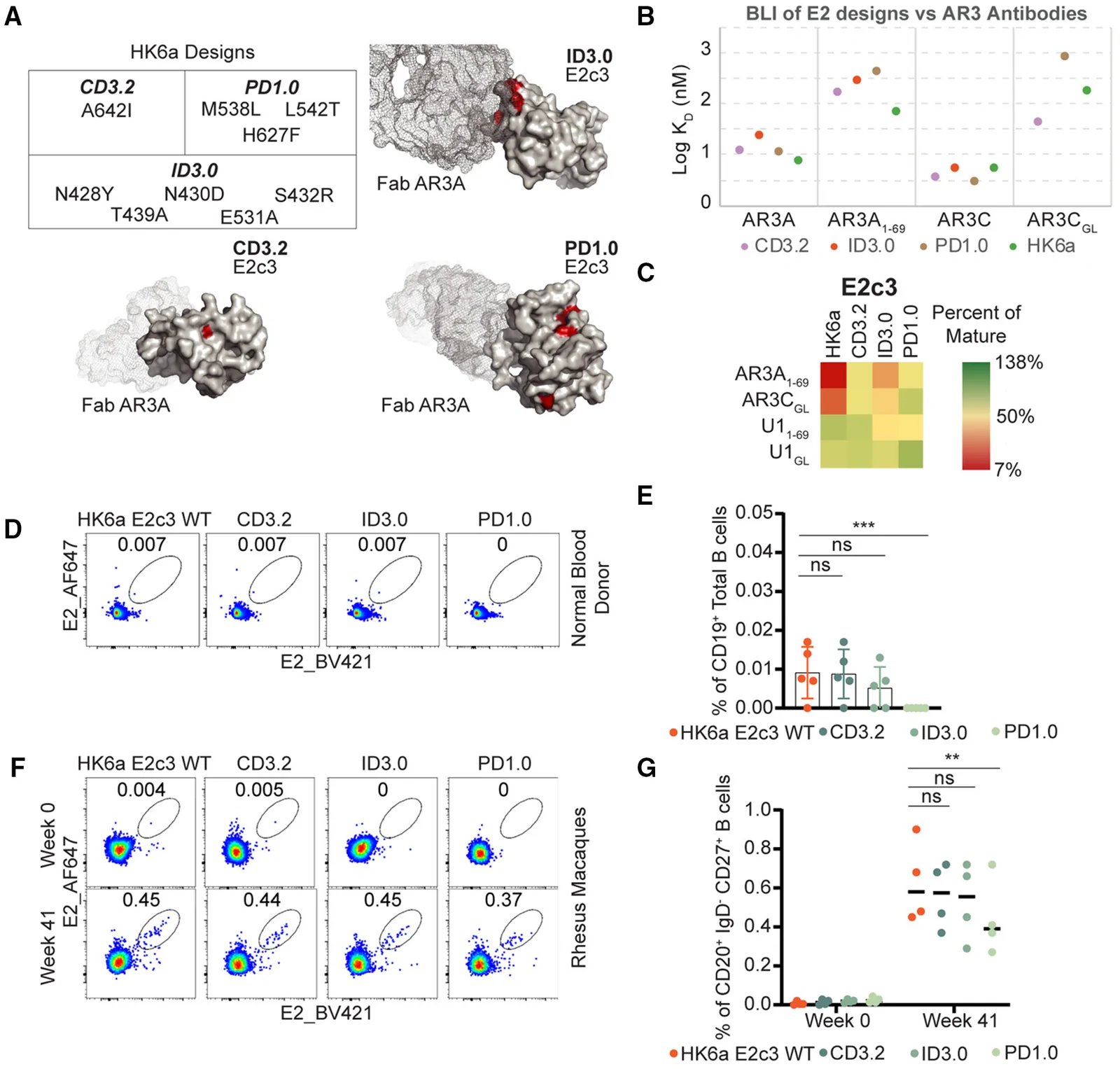
Biochemical characterization of E2 design candidates. **(A)** Structural models of E2 designs based on core mutations CD3.2, interface mutations ID3.0, and PROSS-guided stability mutations PD1.0. Mutation loci are highlighted in red on the surface representation of the protein. **(B)** BLI kinetics of the E2 designs and HK6a with IgG AR3A, IgG AR3A_1-69_, IgG AR3C, and IgG AR3C_GL_. **(C)** ELISA-based cross-reactivity heat map of the HK6a E2c3 wt and designs against mature and precursor Abs. Values represent precursor Ab binding as a percentage of the corresponding mature Ab binding, with the color intensity scale indicated on the right. **(D-G)** Detection of E2-specific B cells in normal blood donors and non-human primates (NHP) immunized with HCV-1 E1E2. Week 0, pre-immunization; week 41, 1 week after the 4^th^ immunization. **(D, F)** Flow cytometry staining of HK6a E2c3 wt, CD3.2, ID3.0, and PD1.0 designs specific B cells. Cells were gated on CD20 + IgD-CD27 + . The data of a representative normal blood donor and NHP are presented. **(E, G)** Frequency of HK6a E2c3 wt, CD3.2, ID3.0, and PD1.0 designs specific B cells in 5 Normal blood donors and 4 NHPs. Statistical significance was determined using two-tailed Mann-Whitney U-tests. **P < 0.005; ns, not significant *P* > 0.05.

In the second protocol, we employed the Protein Repair One-Stop Shop (PROSS) method [55], which integrates structural and evolutionary information derived from multiple-sequence alignments to introduce mutations that enhance protein stability. PROSS has been widely used to improve enzyme stability and to increase the expression of functional proteins. Notably, PROSS has also been applied to the design of antigens to enhance protein stability, including the HIV-1 envelope gp140 and the *P falciparum* invasion protein RH5 [56,57], and the designed variant recently entered clinical trials. More recently, Williams *et al.* used PROSS to engineer stabilizing mutations in the SARS-CoV-2 Spike protein, resulting in improved protein expression and stability while preserving the exposure of neutralizing epitopes [58]. Vaccination studies in mice demonstrated enhanced cross-nAb titers elicited by the PROSS-engineered Spike antigen [58].

Therefore, we generated three cumulative PROSS designs incorporating nested mutations to increase core rigidity and stabilize the overall antigenic complex (PD1.0-1.2) (S4A Fig) and successfully expressed and purified them (S4B Fig). Of these designs, PD1.0 yielded the highest and most homogeneous protein yield and therefore was chosen for downstream functional quantification.

To quantitatively assess the impact of these mutations on Ab recognition, we performed BLI experiments (Fig 4B and S4C Fig). Against mature AR3A, CD3.2 and PD1.0 bound with affinities comparable to wt HK6a E2c3 (KD of 11.8 nM and 11.3 nM, respectively, compared to 7.4 nM for wt HK6a), whereas the interface-stabilization design ID3.0 displayed an approximately three-fold reduction in affinity (KD of 22.7 nM). Notably, profiling across the panel revealed that all three designs engaged AR3C with nanomolar affinities (KD of 2.9-5.4 nM), while retaining measurable binding to the GL precursors AR3A1-69 (KD of 160-424 nM). CD3.2 exhibited some affinity improvement toward AR3CGL (KD of 41.8 nM) compared to the wt HK6a (KD of 173 nM). Subsequent ELISA assays, however, suggested generally improved binding of all three designs to AR3A and AR3C GLs over their mature counterparts relative to the HK6a baseline (Fig 4C), contrasting the BLI results. Based on these biochemical and kinetic profiles, we further analyzed these three designs with downstream antigen-specific B-cell sorting and FACS-based assessment of naïve B-cell recruitment.

### Functional assessment of B-cell recruitment by optimized E2c3 variants

To determine whether our structure-based designs could enhance immune recognition of the HCV E2 antigen, we evaluated their ability to recruit antigen-specific B cells *ex vivo*. We repeated the B cell binding experiments in both normal blood donors and rhesus macaques immunized with HCV-1 E1E2 using the optimized designs, CD3.2, ID3.0, and PD1, and compared their reactivity against the HK6a E2c3 wt (Fig 4D-4G). We observed no significant differences in naïve B-cell engagement between the wt HK6a E2c3 and the engineered variants in either normal blood donors or pre-immunized rhesus macaques (week 0), with all probes exhibiting negligible baseline reactivity. However, following immunization, the designs CD3.2 and ID3.0 showed E2-specific B cell frequencies comparable to those of the wt antigen in rhesus macaques at week 41, one week after the 4^th^ HCV-1 E1E2 immunization. Surprisingly, the PROSS-optimized design PD1.0 exhibited a significant decrease in B-cell binding compared to the wt (P < 0.01) (Fig 4G). These findings indicate that while our computational designs improved the biochemical binding affinity to mature Abs *in vitro*, these improvements did not translate to enhanced B-cell binding in the NHP model. This discrepancy suggests that biochemical affinity alone may be insufficient to overcome the deep-seated GL-binding barriers inherent in the V_H_1-69 lineage Abs.

## Discussion

The clinical observation that approximately 30% of individuals spontaneously clear acute HCV infection provides strong evidence that a protective vaccine is biologically attainable [3]. However, the high rate of viral persistence and the failure of natural immunity to reliably prevent reinfection, particularly among high-risk groups such as PWID, emphasize the urgent need for a vaccine capable of eliciting potent, cross-genotype immune responses [1].

Effective induction of broad and potent neutralizing responses relies on the successful engagement of bnAb-inferred GL BCRs by the priming immunogen [59]. Analyses of naïve B cells from healthy donors and rhesus macaques used in vaccination studies showed minimal recognition of the E2 core antigen, indicating poor engagement of naïve GL BCRs (Fig 1) [50]. These results demonstrate that wt E2 is inherently inefficient at recruiting GL BCRs, highlighting a fundamental challenge in HCV vaccine development. GL-targeting strategies aim to overcome this initial activation barrier by designing immunogens with sufficient affinity to recruit rare or low-affinity precursor B cells. This approach has been validated in HIV-1 vaccine research, where rationally designed immunogens, such as eOD-GT8, have effectively primed VRC01-class V_H_1-2 precursors in human clinical trials. These advancements establish a conceptual framework highly applicable to HCV, where a similar obstacle exists between naïve B-cell recruitment and the development of cross-genotype neutralization. Achieving this goal requires more than understanding the structure of mature bnAb-E2 interactions. It demands a detailed understanding of the GL precursors that trigger these responses [37].

AR3 is a key target for GL targeted structure-based design. Its high sequence conservation and direct overlap with the CD81 receptor-binding site make AR3 an ideal focus for guiding the immune response toward epitopes that are less permissive to viral escape. Moreover, the high plasticity of the AR3 enables binding by various Abs through different binding modes [25,26,38,42,43]. Consistent with this, repertoire analyses of HCV-infected cohorts demonstrate enrichment of AR3-specific V_H_1-69-encoded or V_H_1-69-encoded-like [42,43], emphasizing this lineage as a common immunological solution for targeting the E2 AR3 and the neutralization face. Although other GL pathways have been described [26], V_H_1-69 derived Abs remain the most frequently observed and structurally validated mediators of broad neutralization, suggesting that the V_H_1-69 scaffold offers a genetically and structurally favorable framework for engaging conserved AR3 epitopes. Accordingly, vaccine strategies that effectively recruit and guide this lineage may increase the likelihood of eliciting broadly protective responses, while preserving the capacity to accommodate additional neutralizing trajectories.

Biochemical and functional studies, conducted by us and others (Fig 2), showed that AR3-targeted GL-reverted Abs retain measurable, though reduced, binding to E2, with the degree of reduction varying among Abs. They also exhibit significantly decreased neutralization potency and breadth compared to their mature counterparts. Point mutagenesis analyses of mature Abs further revealed that, for some AR3 bnAbs, decreases in binding and neutralization can be caused by just one or two somatic mutations within CDRH1, such as S30 and S31 [42,43]. Simultaneously, binding and neutralization experiments established that the hydrophobic tip of CDRH2 (residues 53 and 54) is essential for E2 engagement and neutralization by AR3-targeted V_H_1-69 encoded bnAbs [38]. Overall, these findings identify specific molecular determinants that govern GL-to-mature transitions and provide a mechanistic foundation for rational antigen design. To mechanistically contextualize these functional observations, we examined the structural basis of GL Abs engagement with E2.

Structural analysis of HK6a E2c3 in complex with V_H_1-69 GL reverted Fabs, AR3A_1-69_, U1_1-69_, and U1_GL_ (Fig 3), revealed a binding mode that aligns with the typical binding pattern of mature V_H_1-69-encoded Abs (the E2c3-AR3C_GL_ complex will be discussed later). These findings align with the low SHM level and the conservation of interacting residues. A detailed comparison of the E2c3-GL and E2c3-mature Fab complexes showed a similar network of interactions at the Ab-antigen interface (Fig 3C, and S3C Fig), which could be due to the crystal contacts and represents a limitation of X-ray Crystallography. We could not identify a single specific interaction responsible for the decreased binding affinity observed in the GL-reverted Abs. Additionally, although previous mutagenesis studies have shown that reverting CDRH1 residues at positions 30 and 31 to the GL-encoded Serine significantly reduces binding affinity [42,43], our structural analyses suggest that the interactions mediated by these residues are largely preserved in the GL-reverted complexes. A plausible explanation for this difference is that sequence variations between the GL and mature Abs affect the conformational dynamics and stability of the CDR loops, thereby influencing binding energetics without drastically changing the static interaction network observed in the crystal structures. These effects may not be captured in the solid-state environment, where crystallographic constraints can mask subtle differences in flexibility, entropy, or transient contacts that influence affinity in solution.

Notably, the structure of the E2c3-AR3C_GL_ complex revealed a loss of interactions between CDRH2 and E2c3 (S3B Fig), whereas those mediated by CDRH1 and CDRH3 remained largely unchanged (Fig 3A, and S3C Fig). V_H_1-69-encoded Abs possess an unusually hydrophobic CDRH2 [35]. Previous biochemical and structural studies have suggested that this hydrophobic motif initiates engagement with the conserved hydrophobic pocket on E2 [37]. We therefore propose that the apparent loss of CDRH2 interactions in the AR3C_GL_ complex may result from subtle differences in crystal packing, rather than reflecting a fundamental alteration in the binding mode.

Our study provides the first structural characterization of AR3-targeted GL-reverted Abs in complex with E2. Previously, we determined the structures of GL-reverted precursors of the AS412-targeted Abs AP33 and 19B3 bound to an AS412-derived peptide [60]. AS412 is a highly conserved linear epitope located between HVR1 and the E2 front layer (Fig 1A), which includes residues essential for CD81 engagement, such as W420, and is targeted by several well-characterized bnAbs like HCV1, HC33, and AP33 [9]. Although GL-reverted AS412 Abs demonstrated significantly reduced binding affinity, their structures revealed a binding mode and interaction network similar to that of the mature Abs. Overall, these findings further suggest that affinity maturation can enhance binding by altering CDR loop dynamics and stabilizing the paratope.

An essential step in designing GL-targeted vaccines is to engineer an E2 immunogen with increased affinity for GL Abs to better engage naïve B cells. For this purpose, we utilized two Rosetta-based design protocols and applied two complementary methods. The first focused on reengineering E2 to optimize the Ab-antigen interface, thereby directly enhancing interactions with GL-reverted Abs. The second aimed to stabilize the antigen, thereby increasing its rigidity and thus strengthening its interactions with GL-reverted Abs. Our top designs revealed comparable binding ability of the GL Abs relative to that of mature Abs. The lack of enhanced naïve B-cell engagement despite improved biochemical affinity suggests that increasing Ab affinity alone is insufficient to promote productive activation of germline B-cell receptors. Studies of GL-targeting immunogens in the HIV field have demonstrated that successful activation of naïve B cells depends on multiple factors beyond equilibrium affinity, including the frequency of compatible precursor B cells, appropriate heavy- and light-chain pairing, antigen valency and presentation, and the ability to trigger sufficient B-cell receptor signaling for clonal expansion and affinity maturation [61,62]. Consequently, improvements in soluble Ab affinity do not necessarily translate into enhanced activation of membrane-bound B-cell receptors. These observations suggest that future HCV GL-targeting immunogens should consider these additional parameters, together with improvements in antigen stability and presentation, to optimize the elicitation of broadly neutralizing Ab responses.

Collectively, our findings represent an initial step toward defining the structural determinants governing GL recognition by AR3-targeting Abs. Further structural and biochemical characterization of the interactions between E2 and GL reverted Abs, combined with structure-guided immunogen design and functional validation, will be required to optimize antigen configurations that efficiently engage naïve BCRs. Such efforts will be essential for the rational development of vaccine candidates that can reliably initiate and steer bnAb responses against HCV.

## Materials and methods

### Ethics statement

Peripheral blood samples from normal blood donors were collected after written informed consent under a protocol approved by the Institutional Review Board of Scripps Research (IRB#238139).

### Cloning and purification of Abs

DNA of mature and reverted Ab HC variable regions, together with IgG constant regions or the Fab constant region, were cloned into the pCMVtpa vector for mammalian cell expression. The Ab variable regions were ordered as DNA fragments and cloned into the pCMVtpa vector downstream of the secretion signal via Gibson Assembly.

DNA from Ab clones was transfected into HEK293expi GnTI^-^ cells at 1 µg/ml DNA, 3 x 10^^6^ cells/ml, and incubated at 37 °C, 135 rpm, in 8% CO_2_ for 96 hours. Abs were harvested by centrifugation at 500 g, and the media were filtered through a 0,22 µm filter. The media were loaded onto a Protein G column using PBS as the binding buffer and eluted with 100 mM Glycine (pH 2.2). Protein-containing fractions were concentrated and injected into a Superdex 200 Increase 10/300 GL column with a buffer containing 100 mM NaCl and 20 mM Tris-HCl (pH 7.5). Ab purification and correct oligomerization were confirmed by reducing and non-reducing SDS-PAGE and Western blot using HRP-conjugated anti-human Fc Ab. Western blot membranes were developed using ECL.

### E1E2 and E2 cloning

E1E2 cloning - the E1E2 genes (amino acids 192–745 based on the H77 prototypic strain, UniProt number P27958) of the following isolates were cloned into the pCMV vector for mammalian cell expression: H77 [60], HK6a [63], UKNP 1.9.1 (Addgene 97374), UKNP 1.10.1 (Addgene 97376), UKNP 1.11.6 (Addgene 97382), UKNP1.16.3 (Addgene 98188), 1A72 (Addgene 177690), 1a138 (Addgene 177688), UKNP 1.18.1 (Addgene 98190), 1B25 (Addgene 177691), 1B34 (Addgene 177692), 1B58 (Addgene 177693), UKNP 3.1.2 (Addgene 98215), UKNP 4.2.2 (Addgene 98268), and UKNP 5.2.1 (Addgene 98385).

E2c3 cloning - the genes of HCV H77 and HK6a E2c3 (E2 residues 412–645 with an internal truncation of VR2, VR3, and the removal of N448 and N576 glycosylation sites) were cloned into the pCMVtpa vector [37]. The E2c3 constructs of 1b25, UKNP 5.2.1, 1a138, UKNP 4.2.2, and UKNP 1.18.1, as determined by sequence alignments to the H77 and HK6a E2c3, were ordered as DNA fragments from Twist Biosciences and cloned into the pCMVtpa vector downstream of the secretion signal via Gibson Assembly.

E2c3 AviTag cloning - the AviTag was cloned to all pCMVtpa E2c3 isolate plasmids between the C-terminus of the E2 and the stop codon by Gibson Assembly (AviTag sequence – GGCCTGAACGACATCTTCGAGGCCCAGAAGATCGAGTGGCACGAG)

E2c3 design cloning - Synthetic gene fragments (Twist Bioscience) corresponding to five initial core variants were cloned into the phCMV expression vector using Gibson assembly to replace the wt E2c3 sequence. Additional clones were created to facilitate downstream purification by adding a C-terminal Strep-tag II to CD1.0–5.0, placed immediately upstream of the stop codon. For the interface-optimized designs, the wt interface was removed from the phCMV-E2c3 construct and replaced with PCR-amplified fragments containing the desired mutations, generated using hybridized primers. Iterative core and interface variants were generated by site-directed mutagenesis.

### E2 expression and purification

H77 E2c3 and HK6a E2c3 were expressed in stable HEK293T GnTI^-^ cell lines that constitutively express the E2c3 core domain. Cells were maintained under standard culture conditions, and the protein was harvested from the growing media.

For the expression of E2c3 of other isolates and the designed E2c3, DNA was transfected into HEK 293expi F or HEK 293expi GnTI^-^ cells (depending on downstream experiments) at 1 µg/ml DNA, 3 x 10^^6^ cells/ml, and incubated at 37 °C, 135 rpm, in 8% CO_2_ for 72 hours.

E2s were harvested by centrifugation at 500 g, and the media were filtered through a 0.22 µm filter. The media were loaded onto an IgG AR3A-conjugated Protein G column using PBS as the binding buffer and eluted with 100 mM Glycine, (pH 2.2). Protein-containing fractions were concentrated and injected into a Superdex 200 Increase 10/300 GL column with a buffer containing 100 mM NaCl and 20 mM Tris-HCl (pH 7.5). E2 purification was confirmed by SDS-PAGE and Western blot using IgG AP33 as the primary Ab, followed by HRP-conjugated anti-human Fc Ab. Western blot membranes were developed using ECL.

### Strep-tagged E2c3 variants purification

Recombinant proteins were expressed in Expi293F cells as described for E2 expression. Harvested culture media were loaded onto a Strep-Tactin affinity column (GE). The column was washed with 10 column volumes of PBS, and the target proteins were eluted using five column volumes of 2.5 mM desthiobiotin in PBS. Protein-containing fractions were concentrated and injected into a Superdex 200 Increase 10/300 GL column in a buffer containing 100 mM NaCl and 20 mM Tris-HCl (pH 7.5).

### E1E2 lysate expression and purification

HCV E1E2 lysates were expressed by transfection in HEK 293T cells. HEK cells fed with DMEM containing 2% FBS, 1% L-Glutamine were transfected using Polyethylenimine (PEI, Polysciences) at a 3:1, PEI:DNA, ratio (15 µg of DNA for a 15 cm plate, 66% confluency) and incubated for 4 hours at 37 °C with 5% CO_2_. After incubation, cells were refed with DMEM containing 10% FBS, 1% L-Glutamine, and 1% Penicillin/Streptomycin and incubated for 72 hours. Cells were consequently harvested by Trypsin treatment and pelleted at 400 g for 5 mins at 4 °C. Cells were washed with ice-cold PBS and pelleted again. Washed cells were resuspended in ice-cold lysis buffer containing 150 mM NaCl, 50 mM Tris (pH 7.5), 2 mM EDTA, 0.5% Triton, and protease inhibitors (10–20 μL of lysis buffer per 10^^6^ cells) and incubated for 30 mins on ice. E1E2 lysate was clarified by centrifugation at 20,000 g for 15 mins at 4 °C. Supernatant was aliquoted and stored at -80 °C.

### PBMC samples

Peripheral blood samples from normal blood donors were collected after written informed consent under a protocol approved by the Institutional Review Board of Scripps Research (IRB#238139). PBMCs were processed using Lymphoprep and SepMate tubes (StemCell) according to manufacturer’s instructions.

PBMC samples from rhesus macaques were obtained from prior HCV-1 E1E2 mRNA or protein immunization studies (Chen et al, 2020). All animal procedures were conducted in accordance with protocols reviewed and approved by the Institutional Animal Care and Use Committees (IACUC) of Texas Biomedical Research Institute and TSRI. PBMCs were aliquoted and stored in liquid nitrogen.

### E2 antigen probes and flow cytometry analysis

To generate antigen probes for B cell analysis, Avi-tagged E2c3 protein and variants were produced and biotinylated using the BirA biotin–protein ligase kit (Avidity, Aurora, CO) according to the manufacturer’s instructions. Biotinylated antigens were then conjugated to streptavidin–PE (Thermo Fisher Scientific) or streptavidin–BV421 (BioLegend) at a 4:1 molar ratio.

Antigen-specific B cells were analyzed by flow cytometry. Cryopreserved PBMCs were thawed and aliquoted into 96-well U-bottom plates. Cells were first blocked with anti-CD81 Ab (5 μg/mL, BD Biosciences) and Fc receptor blocker (TruStain FcX, BioLegend), then stained with dual-color–labeled antigen probes at 4°C for 30 min. Cells were then incubated with a cocktail of surface markers for an additional 30 min at 4°C in the dark. Dead cells were excluded using 7-AAD, and samples were acquired on a Cytek Aurora equipped with five lasers using SpectroFlo software (Cytek Biosciences). Data were analyzed using FlowJo (BD Biosciences, version 10.9.0). Gating strategies are shown in S1A Fig.

### Biolayer interferometry (BLI)

Binding affinities were measured using BLI (Gator). Assay conditions were first optimized to determine the appropriate loading of purified IgGs onto anti-protein G biosensors, achieving ~70% sensor-binding capacity. BLI measurements were performed in 96-well plates with the following buffer and sample layout: Column 1 (C1), PBS (baseline); C2, IgG loading; C3–C5, PBS; C6–C12, antigen at 0, 20, 40, 80, 160, 320, and 640 nM.

BLI assays were conducted using protein G probes with the following protocol: 60 s baseline (C1), 60 s IgG loading (C2), 60 s baseline (C3), 180 s association (C6–C12), and 120 s dissociation in PBS (C4 for 0 nM antigen; C5 for antigen-containing samples). The equilibrium dissociation constant (K_D_) was calculated as k_Off_/k_On_ All measurements were performed at 25 °C. Binding data were reference-subtracted against the 0 nM antigen control, and local fitting and kinetic analyses were performed using the Gator software.

### Enzyme-linked immunosorbent assay

Half-well high-binding plates were pre-coated with Galanthus nivalis lectin (GNL, Sigma-Aldrich) at 2.5 µg/mL in PBS to enable enrichment of glycosylated E1E2 from cell lysates. Plates were blocked with 5% Bovine Serum Albumin in PBS. After blocking, plates were washed with PBS containing 0.05% Tween-20 (PBS-T) and incubated with a two-fold serial dilution of E1E2 lysate (from 1:1–1:256). Next, plates were washed with PBS-T and tested with IgG AR3A diluted in PBS-T containing 1% Bovine Serum Albumin to determine the optimal dilution for 70% binding. This dilution was used for subsequent experiments with the reverted Abs. Anti-Human Fc conjugated to HRP was used as a secondary Ab, diluted in PBS-T at 1:20000. Detection was achieved using ECL, and the reaction was quenched with 2 M Sulfuric acid after 20 minutes. Absorbance was measured at 450 nm. For pure E2c3, E2c3 was bound directly to the plate (no GNL) by incubation for one hour at 25 °C. All incubation steps were performed at 25 °C for 1 hour or at 4 °C overnight, except for lysate binding, which was performed at 4 °C overnight. All primary Abs were added at a concentration of 5 µg/ml. Reverted Abs ELISAs were performed on the same plate as their mature counterparts and were normalized against the mature Abs. Significant signal for the mature Abs was defined as a minimum five-fold increase in absorbance relative to the negative control. Binding of the reverted Abs was then expressed as a percentage of the corresponding mature Abs signal, allowing for direct comparison across lineages.

### Neutralization assay

HCV pseudoparticles (HCVpp) were produced as previously reported [53]. In summary, HEK293T cells were co-transfected with the pNL4–3.Luc.R^−^ E^−^ plasmid, which encodes HIV-1 Gag-Pol and a luciferase reporter gene (AIDS Reagent #3418), along with HCV E1E2 expression plasmids at a 1:4 ratio using PEI (5:1 PEI:DNA, w/w). Cells were maintained in DMEM supplemented with 10% FBS, sodium pyruvate, L-glutamine, and penicillin–streptomycin, and incubated at 37 °C with 5% CO_2_. After 72 h, supernatants containing HCVpps were collected, filtered through a 0.22 µm filter, and stored at 4°C for up to two weeks or at -80°C for long-term storage.

For the neutralization assay, HuH-7 cells were seeded in 96-well plates (1.5 × 10⁴ per well) and cultured overnight at 37°C and 5% CO_2_. HCVpp stocks were serially diluted and titrated on HuH-7 cells, and infectivity was quantified by measuring luciferase activity to determine the appropriate viral dilution for neutralization assays. For neutralization, HCVpp stocks were mixed with IgGs at concentrations ranging from 5 to 0.001 µg/ml and incubated for 1 h at 37 °C before being added to HuH-7 cells.

After 4–6 h, the medium was replaced with phenol-red-free DMEM, and the cells were incubated for 72 h. Cells were then lysed using a buffer that contained 25 mM glycylglycine, 15 mM MgSO_4_, 4 mM EGTA, 5 mM NaOH (pH 7.8), 0.01% Triton X-100, and luciferase activity was measured using Bright-Glo (Promega). Relative light units (RLU) were normalized to mock controls, and percent neutralization was calculated relative to the signal of HCVpp-only infection.

Half-maximal inhibitory concentrations (IC_50_) were calculated by fitting the normalized neutralization percentages across the concentration range to a non-linear regression model. Specifically, data were modeled using a four-parameter logistic (4PL) sigmoidal dose-response curve with a variable slope. Curve fitting, parameter optimization, and visualization were performed using a custom Python pipeline utilizing the scipy.optimize.curve_fit package. Goodness-of-fit was assessed using the coefficient of determination (R^2^).

### X-ray crystallization

Crystallization trays were set up using the sitting-drop vapor-diffusion method at 20°C. Crystallization screen plates were set up using the Mosquito crystallization system (SPTLabtech) with a protein concentration of 10 mg/ml and JCSG I-IV crystal screens as the reservoir conditions. Crystals of HK6a E2c3-AR3A_1-69_ complex were obtained using a reservoir solution of 0.2 M sodium sulfate and 20% (w/v) PEG 3350. Crystals of HK6a E2c3-AR3C_GL_ complex were obtained using a reservoir solution of 0.1 M HEPES (pH 7.5), 20% (w/v) PEG 4000, and 10% (v/v) isopropanol. Crystals of HK6a E2c3-U1_1–69_ complex were obtained using a reservoir solution of 0.2 M Potassium chloride, and 20% (w/v) PEG 3350. Crystals of HK6a E2c3-U1_GL_ complex were obtained using a reservoir solution of 0.16 M Ammonium sulfate, 0.08 M Sodium acetate (pH 4.6), 20% (w/v) PEG 4000, and 20% (v/v) Glycerol. Prior to data collection, crystals were cryoprotected with 10–15% ethylene glycol or 20% PEG200 (E2c3-AR3C_GL_) and flash-cooled in liquid nitrogen. Diffraction datasets were collected at the European Synchrotron Radiation Facility (ESRF) beamlines ID30-A1 (HK6a E2c3-AR3A_1-69_), ID30-A3 (HK6a E2c3-AR3C_GL_, HK6a E2c3-U1_GL_), and ID23–2 (HK6a E2c3-U1_1–69_), Grenoble, France, at 100 K. The structures were solved by molecular replacement using Phaser, with the E2-mature Ab complex as the search model. Structure refinement and model building were conducted in Phenix [64], and model building was performed with COOT [65]. Final refinement statistics are summarized in S1 Table.

### Buried surface area analysis

Buried surface area at the Ab-antigen interface was calculated using solvent-accessible surface area (SASA) measurements performed in PyMOL [66,67], using a rolling-sphere algorithm with a probe radius of 1.4 Å. The antigen (chain E), heavy chain (H), and light chain (L) were first extracted from the full complex to generate monomeric and pairwise structures. SASA values for the antigen alone were calculated from the isolated antigen structure. To determine chain-specific contributions to antigen binding, SASA values of the antigen were recalculated in the presence of either the heavy chain (E + H complex), the light chain (E + L complex), or the full Ab (E + H + L complex). The buried surface area contributed by each Ab component was computed as the difference between the antigen SASA in isolation and its SASA in the corresponding complex: buried surface area = SASA (antigen alone) - SASA (antigen in complex).

Per-residue buried surface area values for the Ab heavy chain were computed by comparing residue-level SASA values in the free heavy chain and in the antigen-bound complex containing only the heavy chain (E + H). SASA calculations were performed with per-atom surface areas stored in the B-factor field and summed per residue. Residue-level buried surface area was obtained as the difference between the SASA of each residue in the unbound heavy chain and its SASA in the complex. Complementarity-determining region (CDR) contributions were quantified by summing per-residue buried surface area values over standard heavy-chain CDR definitions (CDRH1 residues 31–35, CDRH2 residues 50–65, and CDRH3 residues 95–102, including insertion codes).

### Protein design

Positions in the core of the antigen were determined using the Layer residue selector in Rosetta and designed using FuncLib [54]. Due to the paucity of homologs in certain regions, the calculated Position-Specific Scoring Matrix (PSSMs) in these regions were replaced with a PSSM made using a representative panel of HCV variants [53]. Individual mutations with a PSSM score greater than -2 were modelled on the HK6a E2c3 AR3A_1-69_ and AR3C_GL_ structures, and mutations with Rosetta energy less than 2.5 Rosetta energy units relative to each parental structure were accepted. The resulting sequence space was combinatorially enumerated on both structures, and designs were scored based on the “fuzzy”-logic design approach [68] to bias towards designs that optimized the energies of both structures. Designs were clustered, and five designs with at least six mutations relative to one another were selected for experimental testing.

Positions in the Ab-antigen interface were determined using the InterfaceByVector residue selector in Rosetta. Designs were computed as for the core variants, except that the threshold for allowed mutations was set to 3 Rosetta energy units relative to each parental structure. Designs were clustered, and five designs with at least two mutations relative to one another were selected for experimental testing

The PROSS [55] web server was used to design mutations based on the HK6a E2c3 AR3A, AR3A_1-69_, and the U1_GL_ structure using default settings.

## Supporting information

S1 Fig Structural comparison of AR3-series binding modes. **(A)** Flow cytometry gating strategies for analysis of antigen specific B cells. **(B)** Side-by-side view of the E2 complexes for AR3A (PDB ID: 4MWF) and 212.1.1 (PDB ID: 6WO5). Top - the conformation of the front layer. E2 is shown in surface representation with the CD81 BL in grey and the Front Layer in white. The CD81 BL and the Front Layer are further highlighted in cartoon representation, colored in dark blue and cyan, respectively. Bottom - the position of the CDRHs in the E2c3-Fab structures. The CDRH1, CDRH2, and CDRH3 are shown as cartoons, colored green, violet, and blue, respectively.
(TIF)

S2 Fig Biochemical characterization of the GL-reverted AR3-targeting Abs. **(A)** IMGT V-QUEST predicted GL alleles of the HC (V_H_, V_D_, and J_H_) for four AR3-targeting, V_H_1-69-encoded bnAbs. Due to the HC dominance, the LC was not reverted. **(B)** Multiple sequence alignment of the variable regions of the mature AR3 nAbs and their corresponding GL reversions. **(C)** Affinity chromatography profiles (using IgG AR3A-conjugated Protein G beads) for the purification of H77, HK6a, UKNP 4.2.2, UKNP 1.18.1, 1a138, and UKNP 5.2.1. **(D)** ELISA-based cross-reactivity analysis of mature and GL-reverted Abs against E2c3 of H77, HK6a, UKNP 4.2.2, UKNP 1.18.1, 1a138, and UKNP 5.2.1. Data are presented as a heat map, where binding of GL-reverted Abs is expressed as a percentage of the corresponding mature Ab for each isolate. The color scale is shown on the right. **(E)** BLI binding kinetics of HK6a, UKNP 4.2.1, UKNP 1.18.1, UKNP 5.2.1, and 1a138 E2c3 to the mature and GL-reverted Abs. Sensorgrams were obtained from six two-fold titration series starting at 640 nM, and K_D_ values were calculated using a 1:1 global fitting model (solid line). **(F)** Summary table of kinetic constants (K_D_, K_on_, and K_off_) for the interactions of HK6a, UKNP 4.2.2, UKNP 1.18.1, 1a138, and UKNP 5.2.1 E2c3s with mature and GL-reverted Abs, as determined from BLI. **(G)** Dose-response neutralization curves showing the percentage of neutralization by the mature and GL-reverted Abs against H77 HCV pseudoparticles (HCVpp). Half-maximal inhibitory concentration (IC_50_) values were calculated using Python. IgG U1_1–69_, IgG U1_GL_, and IgG 212.1.1_1–69_ exhibited weak neutralization activity and did not achieve 50% neutralization. Therefore, IC_50_ values could not be determined (N/A, not applicable).
(ZIP)

S3 Fig Structural characterization of mature and GL-reverted Abs. **(A)** Surface representation of mature and GL-reverted Ab-E2c3 crystal structures. All structures are superimposed on the E2c3 of the HK6A E2c3-AR3A complex. E2c3 is color-coded by structural domain: front layer in cyan, beta-sandwich in red, CD81 BL in blue, and back layer in green. **(B)** View of mature (PDB ID: 4MWF) and GL-reverted AR3C CDRH2 interactions with E2c3. **(C)** Schematic contact map summarizing interactions between E2c3 and mature and GL-reverted Abs. The CDRH loop primary sequences are displayed with interactions formed by E2c3 indicated above each sequence. Hydrophobic interactions are shown in green, and polar interactions are shown in blue.
(ZIP)

S4 Fig Biochemical Characterization of E2 Design Candidates. **(A)** Table of total E2 design candidates divided by design method: Core designs (CD), Interface designs (ID), and PROSS designs (PD). **(B)** Size exclusion chromatography profiles of HK6a, CD3.2, ID3.0, and PD1.0 E2c3. **(C)** BLI kinetics of the HK6a E2c3 designs and wt with mature and GL-reverted ARCA and AR3C Abs. **(D)** Flow cytometry staining of HK6a E2c3 wt, CD3.2, ID3.0, and PD1.0 designs specific B cells on week 0 and week 41. Cells were gated on CD20 + IgD-CD27 + .
(ZIP)

S1 Table Data collection and refinement statistics for HK6a E2c3-AR3A_1-69_, HK6a E2c3-AR3C_GL_, HK6a E2c3-U1_1–69_, and HK6a E2c3-U1 _GL_. (PDF)
