# Supplementary material for: Structural and biochemical insights into HCV envelope proteins for germline-targeting vaccines
Source: PLoS Pathog. 2026 Oct 12;22(10):e1014651. doi: 10.1371/journal.ppat.1014651 (PMC13690239; doi:10.1371/journal.ppat.1014651)
Supplement: Supplementary file 1 [file ppat.1014651.s005.pdf]

Table S1. Data collection and refinement statistics for HK6a E2c3-AR3A$_{1-69}$, HK6a E2c3-AR3C$_{GL}$, HK6a E2c3-U1$_{1-69}$, and HK6a E2c3-U1 $_{GL}$.

Data collection	HK6a E2c3-AR3A$_{1-69}$	HK6a E2c3-AR3C$_{GL}$	HK6a E2c3-U1$_{1-69}$	HK6a E2c3-U1 $_{GL}$
Beamline	ESRF ID-30A-1	ESRF ID-30A-3	ESRF ID-23-2	ESRF ID-30A-3
Wavelength (Å)	0.96546	0.96770	0.8731	0.96770
Space Group	$P2_12_12_1$	$P2_12_12_1$	$P2_12_12_1$	$P2_12_12_1$
Unit cell parameters (Å,°)	a=61.4, b=66.9, c=218.7	a=60.3, b=64.4, c=183.3	a=59.8, b=79.0, c=133.5	a=60.3, b=82.0, c=135.9
Resolution range (Å)	46.98-2.04 (2.08-2.11) [a]	44.05-1.93 (2.00-1.93)	54.61-3.30 (3.36-3.30)	45.73-2.75 (2.85-2.75)
Observations	228,946	240,241	98,274	81,061
Unique reflections	57,518 (2,120)	54,095 (5,240)	9,197 (480)	17,730 (8,068)
Completeness (%)	98.8 (98.7)	94.8 (95.3)	92.2 (100)	95.7 (96.7)
I/σ(I)	7.2 (0.7)	7.0 (0.8)	5..1 (0.9)	8.3 (1.2)
$CC_{1/2}$ [b]	0.99 (0.47)	0.99 (0.32)	0.97 (0.33)	0.99 (0.46)
R_{merge} [c]	0.07 (1.09)	0.12 (1.57)	0.68 (8.66)	0.10 (0.93)
R_{pim} [d]	0.05 (0.93)	0.07 (0.89)	0.20 (2.62)	0.07 (0.69)
Redundancy	4.0 (3.8)	4.4 (4.6)	1.7 (1.7)	4.6 (4.8)
Refinement Statistics				
Resolution (Å)	46.98-2.04 (2.08-2.04)	44.05-1.93 (2.00-1.93)	54.61-3.31 (3.42-3.31)	45.73-2.75 (2.85-2.75)
No. reflections	56,082	53,734	8,674	17,703
R_{cryst} [e]/R_{free} [f]	0.235/0.281	0.201/0.245	0.268/0.323	0.236/0.281
Number of atoms				
Protein	4,627	4.661	4,362	4,531
Glycan	70	126	28	41
Water	196	327	15	22
Average B value (Å^2)				
All proteins	66	37	81	72
Glycan	100	65	113	100
Water	62	46	62	67
R.m.s. deviation from ideal geometry				
Bond length (Å)	0.011	0.008	0.013	0.014
Bond angle (º)	1.3	1.2	1.6	1.6
Ramachandran Plot (%) [g]				
Favored	96.6	97.0	95.3	95.0
Outliers	0.3	0.6	0.9	0.5
PDB ID	30BC	30CP	30BG	30BH

[a] Parentheses refer to outer shell statistics.

[b] $CC_{1/2}$ = Pearson Correlation Coefficient between two random half datasets.

[c] $R_{merge} = \Sigma_{hkl} \Sigma_i | I_{hkl,i} - <I_{hkl}> | / \Sigma_{hkl} \Sigma_i I_{hkl,i}$, where $I_{hkl,i}$ is the scaled intensity of the ith measurement of reflection *h, k, l*, and $< I_{hkl}>$ is the average intensity for that reflection.

[d] $R_{pim} = \Sigma_{hkl} (1/(n-1))^{1/2} \Sigma_i | I_{hkl,i} - <I_{hkl}> | / \Sigma_{hkl} \Sigma_i I_{hkl,i}$, where n is the redundancy.

[e] $R_{cryst} = \Sigma_{hkl} | F_o - F_c | / \Sigma_{hkl} | F_o |$ x 100, where F_o and F_c are the observed and calculated structures factors.

[f] R_{free} was calculated as for R_{cryst}, but on a test set of 5% of the data excluded from refinement.

[g] Calculated using MolProbity
